# Study on aluminum nitride/addition-cure liquid silicone rubber composite for high-voltage power encapsulation

**DOI:** 10.1371/journal.pone.0252619

**Published:** 2021-06-01

**Authors:** Zhenzhen Ou, Feng Gao, Lingjian Zhu, Huaijun Zhao, Zihan Xun

**Affiliations:** College of Mechanical and Precision Instrument Engineering, Xi’an University of Technology, Xi’an, China; University of Maryland Baltimore County, UNITED STATES

## Abstract

In view of the development direction of high power and miniaturization of high-voltage power supply, higher requirements are put forward for the breakdown strength, thermal conductivity of packaging materials for its high voltage output module. An electric-insulated heat-conducted material with aluminium nitride as heat conducting filler and addition-cure liquid silicone rubber (ALSR) as matrix for high voltage power encapsulation has been studied. Initially, the thermal conductivity and breakdown strength of composites were explored at different filler fractions. With increase of filler fraction, the thermal conductivity increased and the breakdown strength decreased. Then, with the packaging module volume as the optimization objective and the working temperature as the optimization condition, the temperature distribution of high voltage power supply was studied by using the finite element method, and 40wt% filling fraction was selected as the optimal ratio. Finally, the actual packaging experiment of the high voltage module is carried out. and the variation of the output voltage and temperature with the working time is obtained. According to the experimental results, the output voltage of the high voltage module is basically stable, and the maximum surface temperature is 40.4°C. The practicability of the electric-insulated heat-conducted material has been proved.

## Introduction

High voltage power supply is widely used in industry, medical treatment, scientific research, aerospace and other fields [1–3]. In order to ensure the stability and reliability of output, the insulation package of high voltage output module is very important [4]. Compared with liquid and gas insulating material, solid insulating materials have higher breakdown strength, which can greatly reduce the volume of package module. It is widely used in the occasions with higher volume requirements, such as high voltage power supply for airborne radar transmitter and traveling wave tube [5, 6]. In practical engineering applications, with the increase of volumetric specific power of high voltage power supply, the heat accumulation inside the high voltage packaging module is more and more serious, and its heat dissipation problems have become one of the key factors affecting the performance and life of high voltage power supply [7]. According to Bar-Cohen et al, the stability of electronic devices will decrease by 10% with every 2°C increase in temperature [8]. Accordingly, the insulating encapsulation materials need to have high thermal conductivity apart from high insulation strength, in order to achieve rapid diffusion of heat, reduction of device operating temperature and improvement of output power level [9, 10]. Among polymer materials available for encapsulation, the addition-cure liquid silicone rubber (ALSR) is an ideal encapsulation material since it has no by-product release, less heat generation, as well as small shrinkage during crosslinking. It possesses remarkable properties such as excellent electrical insulation, hydrophobicity, chemical inertness and high tolerance to various radiations, except for a low thermal conductivity (about 0.125~0.25 W (m^-1^ K^-1^)) [11, 12]. Currently, the thermal conductivity of electronic packaging polymers can be enhanced by filling high thermal conductivity and electrical insulating ceramic particles [13–15], such as aluminum nitride (AlN) [16], boron nitride (BN) [17], SiC [18], Si_3_N_4_ [19]. For example, Yang et al proposed to modify the surface of AlN powder by atmospheric pressure plasma method. Using modified AlN as filler and polydimethylsiloxane (PDMS) as matrix, AlN /PDMS composite samples were prepared. According to the experimental results, the thermal conductivity of the composites has a nonlinear proportional relationship with the concentration of modified AlN powder. The thermal conductivity of the composite with 75wt% modified AlN /PDMS composite is 1.60 times of that of the unmodified AlN/PDMS composite [20]. But for ALSR, the research mainly focuses on the tracking resistance, flame retardancy and thermal degradation mechanism [21, 22]. The effect of filled ceramic particles on the thermal conductivity and breakdown strength of ALSR is less studied. Moreover, in the practical application of polymer composites, high thermal conductivity and high breakdown strength are often mutually exclusive [23, 24]. For example, Yu et al. provided a method to prepare thermally conductive and electrically insulating BN/cellulosic fibre composites. The thermal conductivity of the composite reached 0.682 W (m^-1^ K^-1^) that increased by 387% with h-BN loading of 41.08 wt%. But the breakdown strength of the composite is only 9.2kV/mm[25]. This is mainly because the mechanism of filling thermal conductive particles to enhance the thermal conductivity is to form the thermal conductive network inside the composite material [26]. The higher the filling fraction is, the more the thermal conductive network is formed and the greater the thermal conductivity is. But for breakdown strength, the formation of thermal conduction network may lead to an increase in leakage current, thus resulting in a decrease in breakdown strength [27]. The decrease of breakdown strength of composite will lead to the increase of insulation layers thickness and enlarged volume of high voltage power supply, which is contrary to the development trend of miniaturization. Therefore, in practical applications, it is very important to select the appropriate ratio of composite insulation thermal conductive materials according to the working temperature, insulation voltage and cost performance, so that the reliability and stability of high voltage power supply can be guaranteed while realizing high frequency miniaturization.

In this work, commercially available aluminum nitride was investigated as thermal filler to prepare ALSR composites. Initially, the variations of thermal conductivity and breakdown strength of the composites with different filling fraction were investigated. Then the temperature distribution of composite materials used in high voltage power insulation packaging is studied by using finite element method, and the optimal composite material ratio is selected with volume as the optimization objective. Finally, the actual high voltage module is packaged, and the variation of output voltage and module temperature during steady state operation is measured to verify the feasibility and applicability of the prepared materials.

## Materials and methods

### Materials

Vinyl-terminated polydimethylsiloxanes (Vi-PDMS, viscosity of 1000 cSt, vinyl content of 0.82 mol%), Polymethylhydrogensiloxane (PHMS, viscosity of 20~30 cSt and hydrogen group content of 0.3wt%) were supplied by Zhejiang Jiande polymerization New Material Co., Ltd, China. Platinum (0)-1,3-divinyl-1,1,3,3-tetramethyldisiloxane complex solution (Karstedt’s catalyst, platinum content of 3000 ppm), 1-Ethynyl-1-cyclohexanol (inhibitor), were purchased from Sinopharm Chemical Reagent Co., Ltd., China. Aluminum nitride (relative dielectric permittivity of 4.0, thermal conductivity of 260 W (m^-1^ K^-1^); average sizes of 1μm, density of 3.26 g/cm^3^) was provided by Shanghai Cwnano Technology Co., China. Fumed nano-silica (anti-settle agent, average sizes of 7nm, hydrophobic) was provided by Evonik Industries AG, Germany.

### Sample preparation

The composites were prepared by mixing VI PDMS and PHMS with a mechanical stirrer at 750 rpm for 10 min, and the molar ratio of Vi-PDMS to PHMS was satisfied with n_Si-Vi_: n_Si-H_ = 1:1.2. Then AlN and fumed nano- silica were dispersed in the mixture successively and stirred mechanically for 150min. After that, the inhibitor was added to the mixture and stirred for 10 min. Then the Karstedt’s catalyst was added and stirred for 10 min as well. After the stirring is over, the mixture was poured into stainless-steel molds for vacuum deaeration until all air bubbles disappear. Finally, the stainless-steel mould was placed in an oven at 120°C for 150 minutes to achieve curing.

### Characterization

The thermal conductivity of the samples was measured based on the laser flash method, and it can be calculated by the equation: Tc = λ× C_p_ × ρ. Where λ is the thermal diffusivity, C_p_ is the specific heat capacity, and ρ is the density of the sample. The density (ρ) was measured based on the Archimedes principle. Thermal diffusivity (λ) and specific heat capacity (C_p_) was measured by flash thermal diffusivity instrument (LFA467, Netzsch Instruments Co.) at room temperature. The used samples are cylindrical with 10mm diameter and 1.5~2 mm thickness.

Dielectric breakdown strength was measured based on ASTM D 149 standard. The sample is square with a side length of 100mm and a thickness of 1.5~2mm. The spherical-spherical electrode system was used to ensure that the electric field at the edge of the electrode does not change too much. The diameter of the electrode is 25 mm. The sample was inserted between two sphere electrode and were immersed in transformer oil together with the electrodes to enhance the surrounding insulation. The fast boost mode was used in the experiment. The test voltage of 50Hz was provided by a transformer and boosted by the rate 0.5 kV/s until the breakdown occurred.

The fracture surface morphology of the composites was observed by scanning electron microscopy (SEM, MERLIN Compact, ZEISS) after being coated with gold.

## Results and discussion

### Thermal conductivities of composites

**Fig 1:**
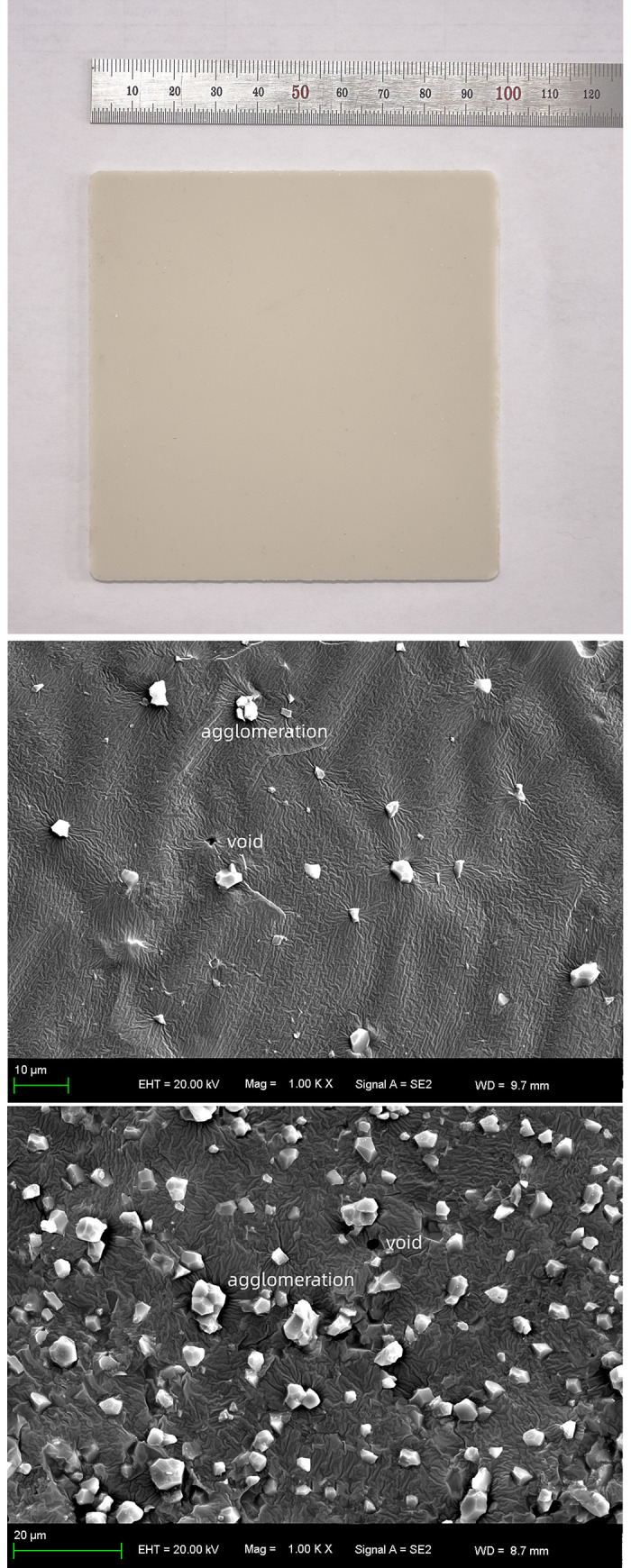
Actual picture of the composites with 10% filling fraction (a), SEM images of the composite with 10% filling fraction (b) and 40% filling fraction (c).

**Fig 2:**
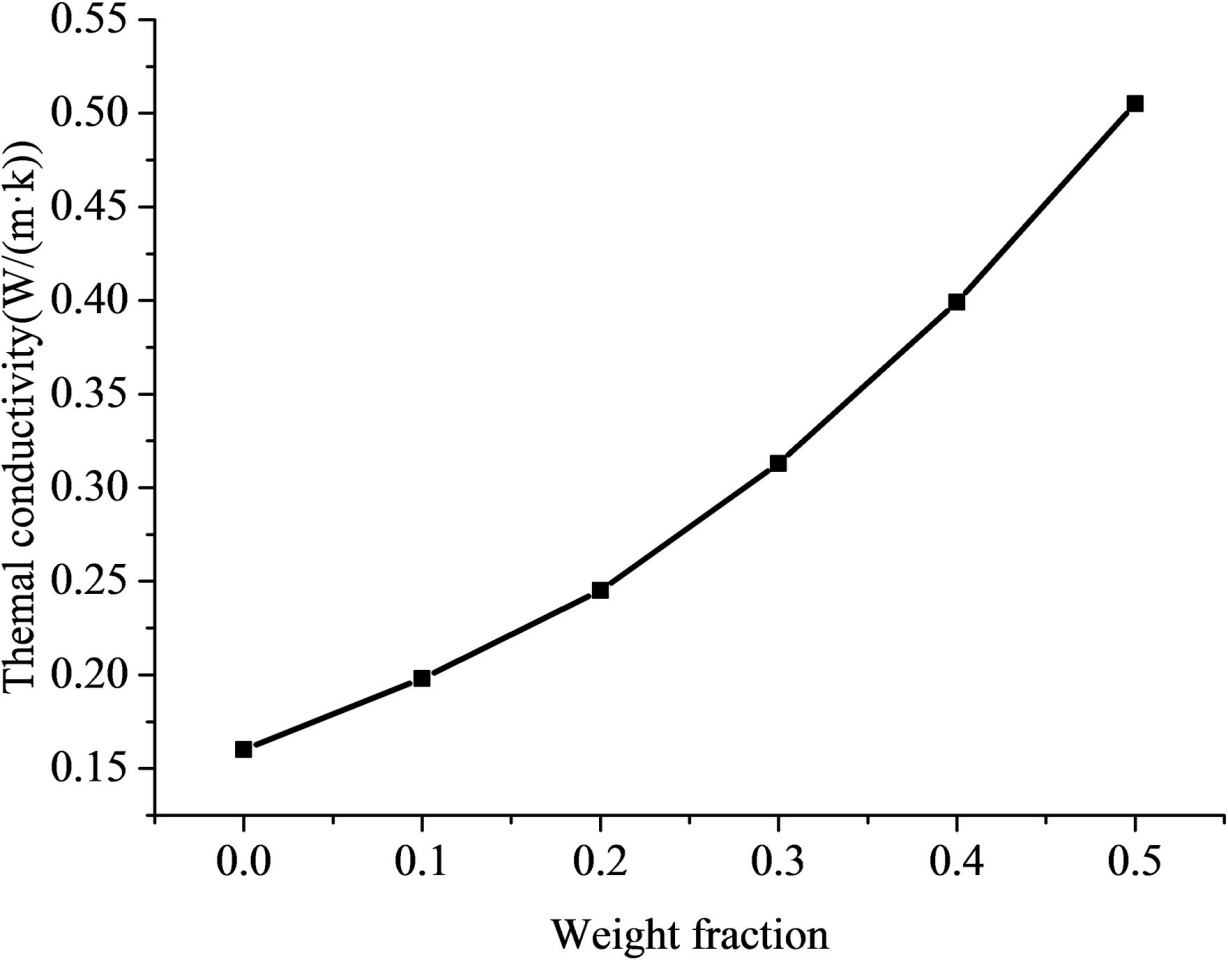
Thermal conductivity of AlN/ALSR composites with different filler content.

The different mass fraction of AlN fillers to affect the thermal conductivity enhancement of ALSR under the same conditions was studied firstly. The actual picture and SEM images of the AlN/ALSR composites were shown in Fig 1. The density and heat capacity of AlN /ALSR composites with different filler contents were shown in Table 1. And the thermal conductivity coefficient was shown in Fig 2.

**Table 1:** Density and heat capacity of AlN/ALSR composites with different filler content.

Weight fraction	0	10%	20%	30%	40%	50%
**Density (g/cm**^**3**^**)**	1.03	1.1	1.19	1.31	1.43	1.57
**Heat capacity(J/g/K)**	1.468	1.406	1.375	1.364	1.348	1.329

It can be seen from the Table 1 and Fig 2 that with the increase of AlN filler content, the heat capacity of the composites decreases, and the density and thermal conductivity of the composites increase. When the filling mass fraction is 50wt%, the thermal conductivity coefficient of AlN/ALSR composites is 0.505 W (m^-1^ K^-1^), which is 3.16 times of that of pure ALSR (0.16 W/(m·K)). It also can be observed that at lower filler loading, the thermal conductivity of AlN/ALSR composites is less increased. This is due to the fact that AlN particles are randomly dispersed in ALSR matrix at this time (Fig 1B), there is no contact between them and the heat conduction chain cannot be formed. The thermal conductivity of fillers does not contribute much to the thermal conductivity of ALSR composites. The thermal conductivity of the composite is mainly determined by the polymer matrix itself. With the increase of AlN filler content, the probability of contact between the particles increases (Fig 1C), and effective heat transfer path of AlN might be formed, which decreases the thermal contact resistance and increases the thermal conductivity significantly [28, 29].

### Dielectric breakdown strength

**Fig 3:**
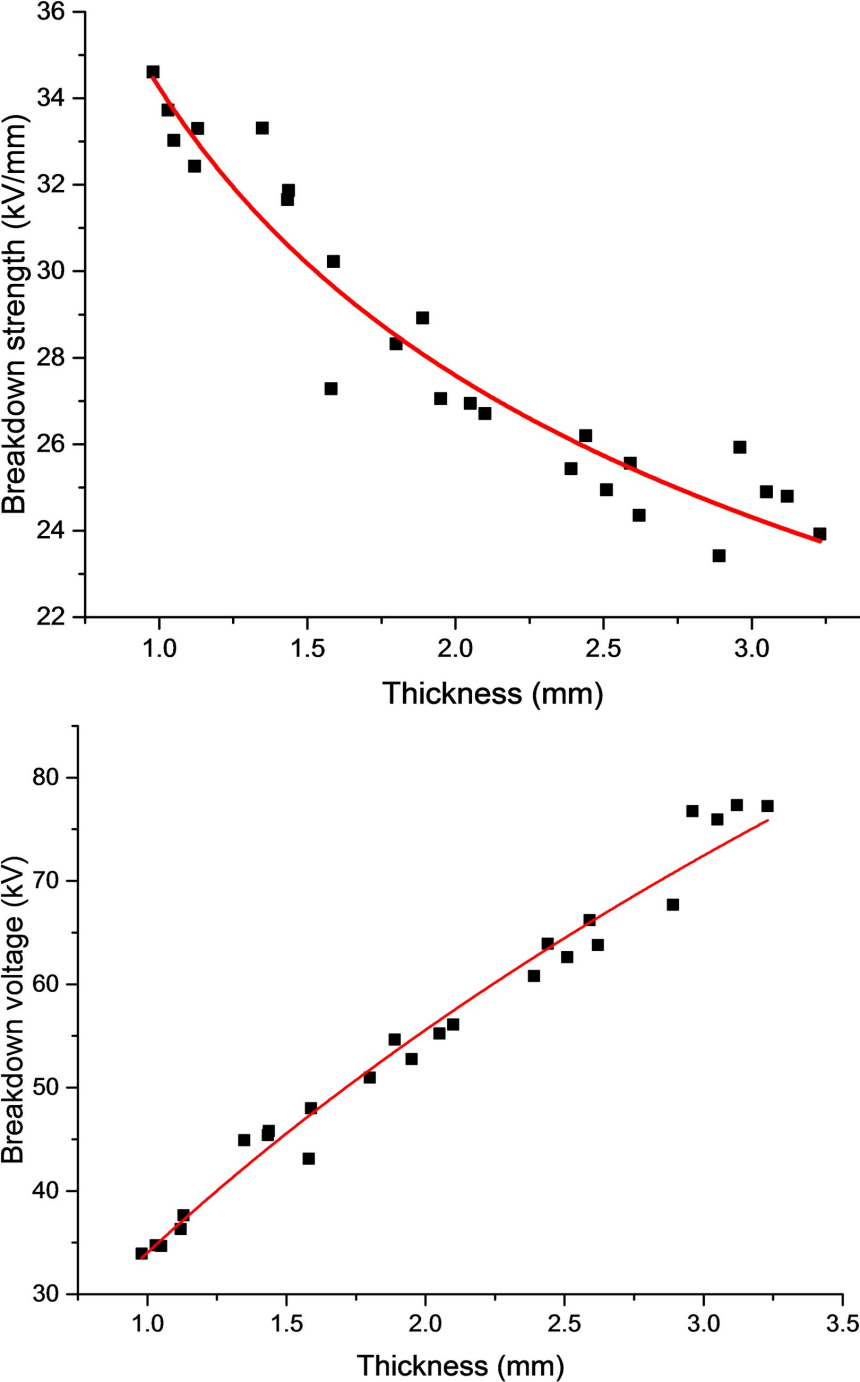
Fitting curve between breakdown strength and thickness (a), breakdown voltage and thickness(b) of pure ALSR.

As suggested in the existing literature, the breakdown voltage is directly proportional to the sample thickness when the solid dielectric thickness is within a 0.01~1 mm range [30, 31]. In other words, the breakdown strength is irrelevant to the sample thickness. After the solid dielectric thickness exceeds 1 mm, the breakdown voltage has a nonlinear relationship with the sample thickness. For pure ALSR, measurement of breakdown strength and breakdown voltage was performed within a thickness range of 1–3 mm, and the results are displayed in Fig 3. As is clear, the breakdown strength (y_1_) and the thickness (x) were approximately logarithmic, and fitting yielded:

$$y_{1}=e^{\left(-0.312lnx+3.5337\right)}$$ (1)

Then the expression of breakdown voltage (y_2_) and sample thickness is as follows:

$$y_{2}=x*e^{\left(-0.312lnx+3.5337\right)}$$ (2)

According to Eqs (1) and (2), the characteristic curves for breakdown strength and breakdown voltage variations with thickness were derived, which are presented in Fig 3 as well. As can be seen: (1) Within a 1.0–3.0 mm thickness range, the breakdown strength of the ALSR composites tended to decrease, which slowed down gradually with increasing thickness. This was attributed primarily to the increased probability and number of tiny defects inside the samples with increasing sample thickness. Nevertheless, the factors relating to such influence were not static. As the thickness increased, the influence factors diminished progressively, which was reflected as the decelerating decline of breakdown strength. (2) Within a 1.0~3.0 mm thickness range, the breakdown voltage of the composite samples tended to rise, despite at a decelerating rate as the sample thickness increased. The curve sustained a certain saturation, which was consistent with the fact that the breakdown voltage could not increase indefinitely with increasing thickness.

**Fig 4:**
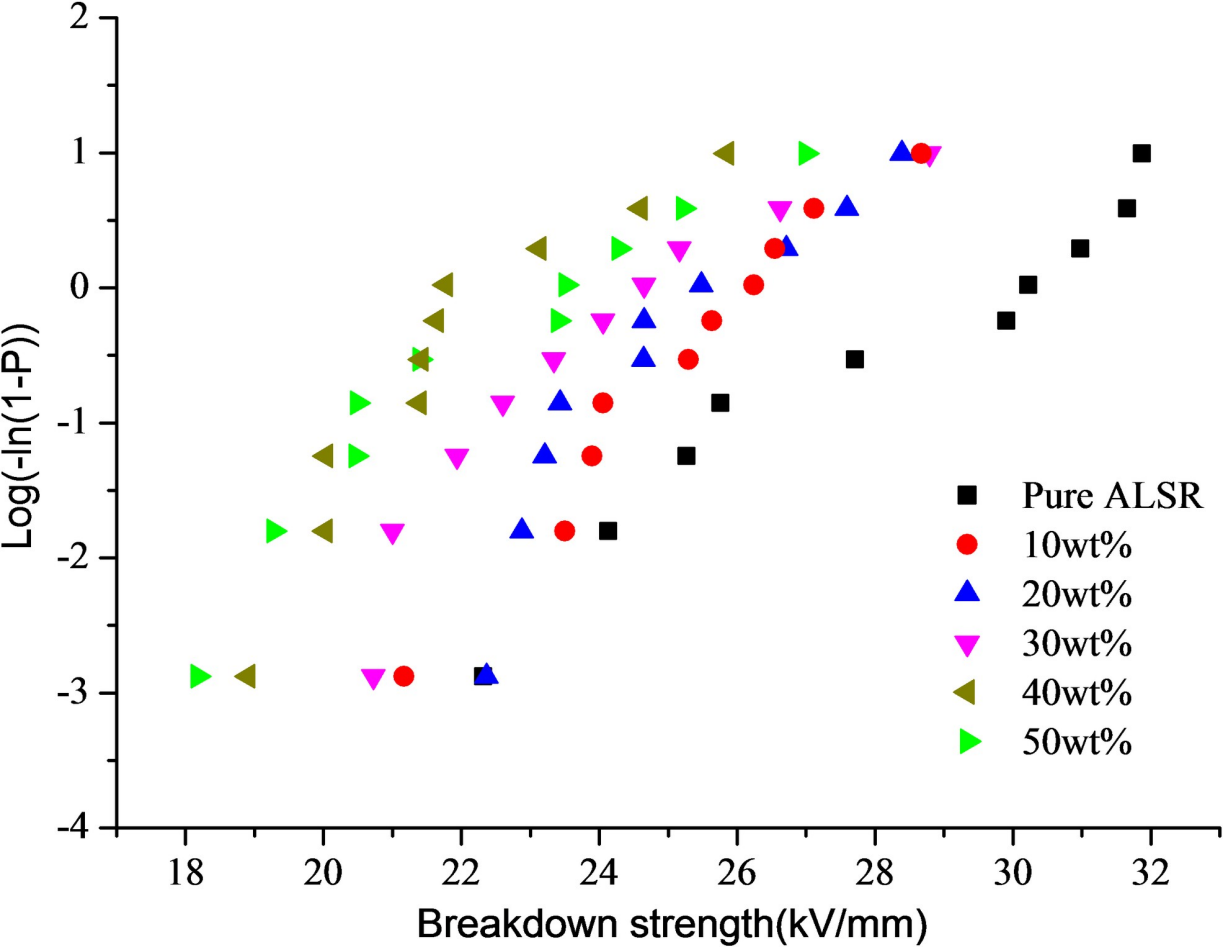
Weibull distribution of breakdown strength.

After obtaining the fitting relationship between the breakdown strength and the sample thickness, according to IEEE Std 930, the breakdown strength of the composite samples was analyzed by using the two parameter Weibull statistical distribution equation. The Weibull plot for the experimental data is shown in Fig 4, and the corresponding Weibull parameters are summarized in Table 2. Where the β represents the dispersion of the failure data, and the α represents the value of breakdown strength in kV/mm when the cumulative failure probability is 63.2%. Furthermore, the correlation coefficient is calculated as a measure of the quality of the Weibull distribution. It can be seen that after the addition of the AlN filler, the breakdown strength of the composite sample is lower than that of the ALSR matrix and decreases as the filling fraction increases. This is mainly attributed to the introduction of inorganic fillers into ALSR matrix, which increases the number and probability of free carriers participating in the conduction, and forms percolation network and conductive path in the composite. So that electrons can move along the nearest particle interface with little energy acceleration from the external electric field, resulting in the decrease of breakdown strength of the composite[32]. In addition, the electric field around the filler may be distorted due to the different dielectric constants of the filler and the ALSR matrix, resulting in the decrease of the breakdown performance of the composites [33, 34]. With the increase of filler mass fraction, not only the number of defects introduced increases, but also the distance between ceramic fillers decreases, which will be more conducive to the transmission of charge carriers and accelerate the breakdown process.

**Table 2:** Weibull parameters.

Sample type	scale parameter (α)	shape parameter (β)	Correlation coefficient
**Pure ALSR**	29.580	9.216	0.977
**10wt%**	26.446	19.039	0.959
**20wt%**	25.833	11.827	0.936
**30wt%**	24.939	9.518	0.948
**40wt%**	23.542	8.301	0.973
**50wt%**	22.736	10.512	0.940

## Simulation and experiment

### Finite element analysis

**Fig 5:**
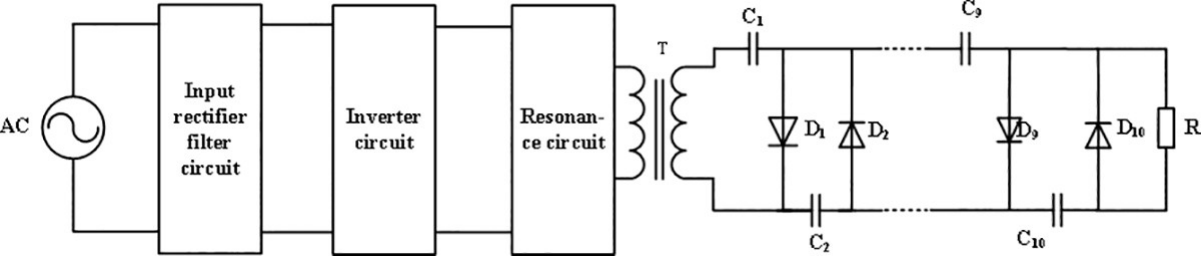
Topological structure of high voltage power supply.

By measuring the density, thermal conductivity and breakdown strength of the composites, it can be seen that with the increase of filler, the density and thermal conductivity of the composites increase, while the breakdown strength decreases, and the high thermal conductivity and high breakdown strength are mutually exclusive, which is consistent with the previous literature. When applied to the high-voltage power supply electronics encapsulation, although the increase in filler would lower the module device temperature, it would also lead to thickened encapsulation layer, as well as increased high-voltage power supply weight and volume. Given the development trends of high-voltage power supply towards high-frequency miniaturization and low weight, it is necessary to comprehensively consider the thermal conductivity and breakdown strength of composite materials, that is, composite materials with high breakdown strength should be selected as far as possible under the condition that the heat dissipation meets the working temperature of internal components of the encapsulation module. On the basis of the above research, taking the volume of high voltage output module as the optimization objective and the internal temperature as the optimization condition, the ratio parameters of AlN / ALSR composites were optimized by finite element analysis of temperature field. The design specifications for high-voltage power supply were as follows: the output voltage was set at 40 kV ~100 kV, the output power was 3 kW, and the operating frequency 100 kHz. The circuit topology is shown in the Fig 5, in which the high voltage output module is composed of Cockcroft-Walton (C-W) 10 stage voltage doubling rectifier circuit. According to the design index, the maximum working voltage across capacitors, the maximum reverse working voltage of diodes were both 20kV, and the maximum potential relative to the ground is 100kV. Eventually, we adopted high-voltage diodes with a diameter of 8 mm and a height of 40 mm, which had a repetitive peak reverse voltage of 30 kV and an average output current of 500 mA. Meanwhile, 2200pF capacitors having a withstand voltage of 30 kV were used, which were 23.2 mm in diameter and 10.3 mm in height. In the voltage- doubling rectifier circuit, all the high-voltage diodes and capacitors served as the heat sources. Circuit simulation and analytical computation found that under steady-state operation, the average heat consumption was 0.36W for the high-voltage capacitor, while was 0.55 W for the high-voltage diode. Besides, the high-voltage capacitor had operating temperatures ranging from -30°C to +85°C, whereas the diodes had operating junction temperatures ranging from -40°C to +175°C.

The finite element analysis model of thermal field for voltage doubling rectifier circuit is established firstly. The dimensions of substrate are 235.5mm × 72mm × 1.6mm, spacing between diodes or capacitors in the same rows was 48 mm, and the spacing between two rows was 58 mm. Then the AlN/ALSR composites with different fraction were estimated for thickness during encapsulation of the rectifier module, which was based on the breakdown strength and maximum potential measurements. Due to the experimental limitations, we didn’t get the fitting formulas for the breakdown strength and voltage variations with sample thickness for each mass fraction. Hence, the encapsulation thicknesses of various AlN/ALSR composites were derived based on the obtained fitting formula for breakdown strength of pure ALSR, as well as the breakdown strengths of ALSR composite samples within a 1.5~2 mm thickness range. Meanwhile, the encapsulation thickness of pure ALSR under maximum potential was derived to be 4.747 mm according to the relevant breakdown voltage fitting formula. At this point, the breakdown strength was calculated to be 21.068 kV/mm, which was 0.712 times that of the pure ALSR (29.58 kV/mm) within a thickness range of 1.5~2 mm. In a similar way, the encapsulation thicknesses of ALSR composites were derived at various mass fractions. To ensure sufficient insulation strength, the calculated theoretical thicknesses were multiplied by a factor of 1.5. Table 3 lists the insulating layer thicknesses at various filling fractions.

**Table 3:** Encapsulation module parameters.

Mass fraction	Breakdown strength (kV/mm)	Thermal conductivities (W/(m·K))	Potting thickness (mm)	Module volume (mm*mm*mm)
**0**	29.580	0.16	7.12	249.74*86.24*39.34
**10**	26.446	0.198	7.96	251.42*87.92*41.02
**20**	25.833	0.245	8.16	251.82*88.32*41.42
**30**	24.939	0.313	8.45	252.4*88.9*42
**40**	23.542	0.399	8.95	253.4*89.9*43
**50**	22.736	0.505	9.26	254.02*90.52*43.62

**Fig 6:**
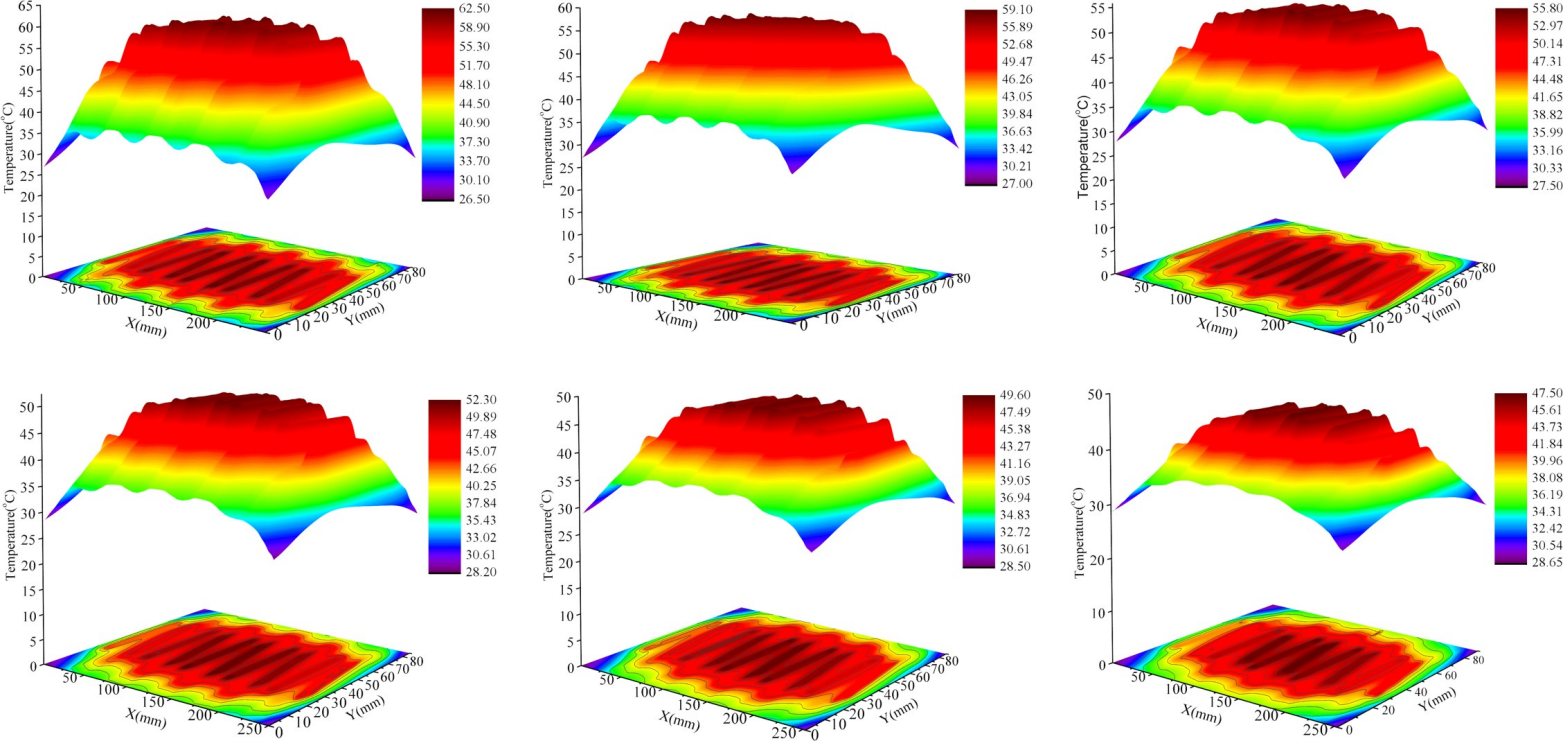
Three-dimensional temperature nephogram of pure ALSR (a), 10wt% AlN/ALSR composites (b), 20wt% AlN/ALSR composites (c), 30wt% AlN/ALSR composites (d), 40wt% AlN/ALSR composites (e) and 50wt% AlN/ALSR composites (f).

According to the above calculated package thickness, ANSYS Icepak is used to analyze temperature field of the voltage doubling rectifier module. The thermal conductivity, heat capacity and density of composites with different fraction are substituted. The temperature distribution cloud diagram and isotherm are shown in the Fig 6. From Fig 6, it can be seen that (1) With the increase in filler mass fraction, the maximum temperature inside the encapsulated module dropped in a gradual manner. At a mass fraction of 50wt%, the maximum temperature was 15°C lower than that during pure ALSR encapsulation, which was attributed chiefly to the increased thermal conductivity and encapsulation volume. (2) As the filler fraction increased, the temperature distribution of module became increasingly uniform. For example, the temperature distribution range of pure ALSR is 26.5°C ~ 62.5°C, while the temperature range of composite with 50wt% filling fraction is reduced to 28.65°C ~ 47.5°C. (3) With the increase of filler fraction, the extent of the temperature drop gradually decreases, which indicates that the efficiency of this method of reducing module temperature by increasing the mass fraction of filler to improve the thermal conductivity of composite materials will be lower and lower. But it is well known that the cost of potting materials will increase with the increase of filler fraction. Therefore, choosing the appropriate proportion of potting materials can not only solve the contradiction between insulation, heat dissipation and module volume, but also improve the cost performance.

Considering the influence of temperature on the lifetime of diodes and capacitors, that is, the lifetime of electronic devices will decrease by 10% when the temperature increases by 2°C, the temperature of electronic device should not be higher than 50°C in actual operation. Based on the above analysis, the maximum temperature was 49.6°C in the case of the composite with 40wt% fraction, which conformed to the encapsulation conditions of the voltage- doubling rectifier module.

### Experimental analysis

On the basis of the above research, the composite material with mass fraction of 40wt% was used as the encapsulation material to encapsulate the actual voltage doubling rectifier module of high voltage power supply. The variation of output voltage and surface temperature with time was measured to verify the feasibility and applicability of the composite material in the field of high voltage power supply packaging.

**Fig 7:**
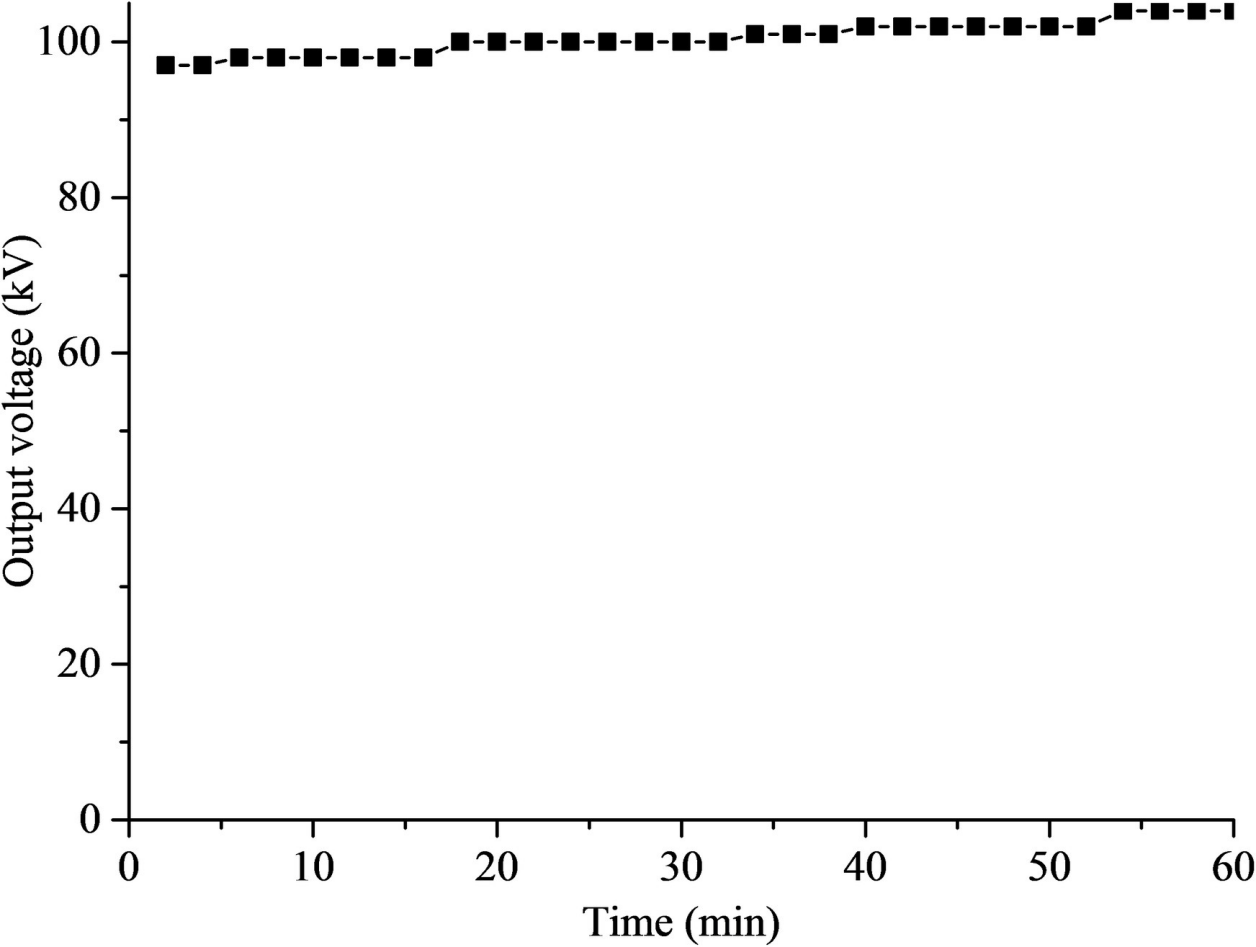
Variation of output voltage.

The output voltage variation of high voltage power supply is tested by high voltage voltmeter. The theoretical output voltage is set to be 100 kV. After power on, the stable output can be quickly realized. The output voltage is measured every 2 min. Fig 7 is the variation curve of output voltage within an hour. It can be found that the output voltage increases slightly with the increase of operation time. After the high-voltage power supply runs for 60 min, the output voltage is 105 kV, and the error with the theoretical output is 5%, which is within the acceptable range. Therefore, the insulation strength of the composite meets the requirements.

**Fig 8:**
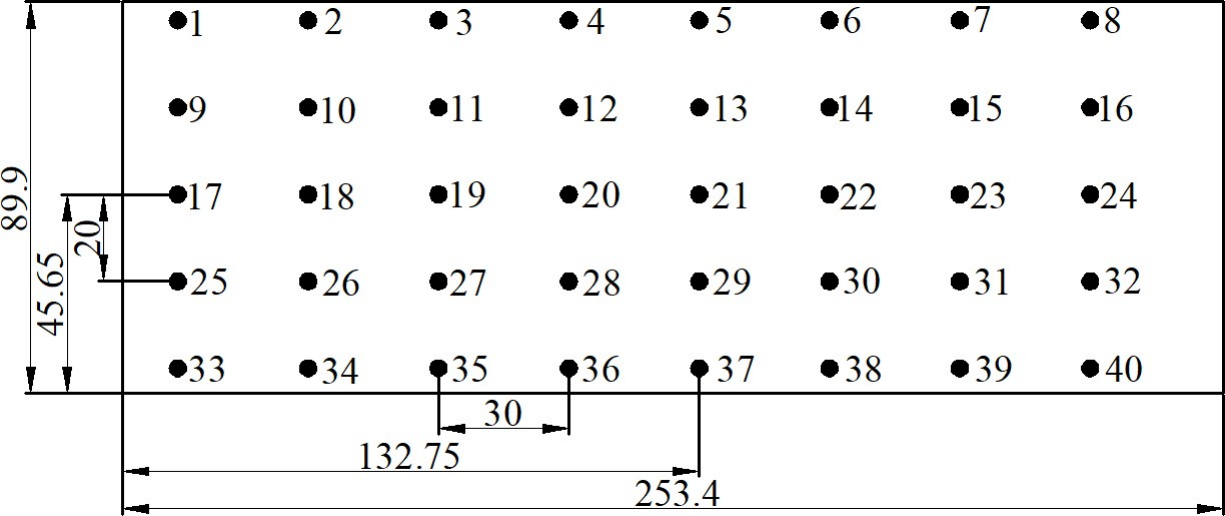
Distribution of temperature measurement points.

Then, the surface temperature variation of the voltage-doubling rectifier module was studied. According to the temperature distribution on the rectifier module upper surface at an encapsulation material mass fraction of 40%, the temperature peak was located at the point 21 (45.65, 132.75). Centering on this point, measuring points were arranged at horizontal intervals of 30 mm and vertical intervals of 20 mm for temperature measurement. Fig 8 depicts the final distribution of temperature measuring points.

The ambient temperature and surface temperature variations of rectifier module was monitored during operation by utilizing infrared thermometers. Record the temperature value every 5 minutes. I According to the measurement results, it can be found that the ambient temperature changes are less than 0.1°C and the temporal temperature changes on the module surface is less than 1°C after 30 minutes, suggesting a state of thermal equilibrium between the voltage doubling rectifier module and the surrounding environment at that time.

After working for 60 minutes, the ambient temperature around the voltage doubling rectifier module increased from 21.9°C of the initial time to 25.1°C. And the comparison between the actual measured temperature and simulation temperature at various measuring points on the two opposite surfaces are shown in the Table 4. As can be seen from Table 4, no matter the upper surface or the lower surface of the voltage doubling module, the distribution of the actual measured temperature is consistent with the simulation results, and generally smaller than the simulation results. The maximum error was 6.13%. Major causes of the errors between actual structure and simulation were analyzed as follows: (1) Infrared thermometers themselves had measurement errors. (2) Diode and capacitor simulation models inside the voltage-doubling rectifier module were somewhat simplified for improving the simulation efficiency, which differed from reality. (3) Although the device power consumption was constant during simulation, it would change in actual operation due to temperature variations. (4) There is an error between the actual size of the doubling module and the theoretical size. In addition, by comparing the actual measurement results of the upper and lower surfaces, it can be found that the temperature of the lower surface is 0.1°C ~ 2.4°C higher than that of the upper surface. This is mainly because the lower surface is closer to the heating device inside the voltage doubling rectifier module. It has been proved that composite materials can achieve effective heat dissipation. In conclusion, it can be considered that the simulation results are credible. The thermal conductivity of the composite with 40wt% filling fraction can meet the heat dissipation requirements of high voltage output modules, and the composite can be applied to high voltage power supply packaging.

**Table 4:** Experimental measurement temperature.

Temperature measuring point	Upper surface			Lower surface		
	Simulation temperature (^o^C)	Actual measurement data (^o^C)	Error (^o^C)	Simulation temperature (^o^C)	Actual measurement data (^o^C)	Error (^o^C)
**1**	29.3	28.3	3.41%	29.4	28.8	2.04%
**2**	33.6	31.7	5.65%	33.5	31.8	5.07%
**3**	34.6	32.6	5.78%	34.9	33.0	5.44%
**4**	35.1	34.1	2.85%	35.5	35.0	1.41%
**5**	35.6	35.0	1.69%	35.9	34.9	2.79%
**6**	34.9	33.9	2.87%	35.3	34.2	3.12%
**7**	34.5	33.4	3.19%	34.7	33.6	3.17%
**8**	32.8	31.3	4.57%	32.6	31.5	3.37%
**9**	32.5	31.8	2.15%	34.4	32.5	5.52%
**10**	37.8	35.9	5.03%	39.4	37.2	5.58%
**11**	39.5	37.5	5.06%	41.1	38.7	5.84%
**12**	40.3	38.5	4.47%	42.4	39.8	6.13%
**13**	40.9	39.9	2.44%	42.4	40.5	4.48%
**14**	40.0	39.2	2.00%	42.0	40.4	3.81%
**15**	39.3	38.0	3.31%	41.1	38.9	5.35%
**16**	36.5	34.4	5.75%	37.9	35.6	6.07%
**17**	33.7	32.9	2.37%	36.2	34.4	4.97%
**18**	38.6	36.5	5.44%	41.3	38.9	5.81%
**19**	41.0	38.5	6.10%	42.3	39.9	5.67%
**20**	42.0	40.2	4.29%	43.5	41.1	5.52%
**21**	42.3	40.9	3.31%	44.0	42.1	4.32%
**22**	41.8	40.3	3.59%	43.5	41.4	4.83%
**23**	40.3	39.1	2.98%	42.7	40.8	4.45%
**24**	37.1	35.8	3.50%	39.5	37.1	6.08%
**25**	32.5	31.1	4.31%	34.3	32.2	6.12%
**26**	37.9	35.6	6.07%	39.0	36.7	5.90%
**27**	39.4	37.3	5.33%	41.3	38.8	6.05%
**28**	40.0	38.4	4.00%	41.7	39.9	4.32%
**29**	40.9	39.7	2.93%	42.2	40.2	4.74%
**30**	39.8	38.7	2.76%	41.7	39.7	4.80%
**31**	38.9	37.5	3.60%	40.2	38.3	4.73%
**32**	36.2	34.5	4.70%	37.5	36.0	4.00%
**33**	29.2	28.8	1.37%	29.2	29.0	0.68%
**34**	33.4	31.5	5.69%	33.1	31.9	3.63%
**35**	34.3	32.4	5.54%	34.4	33.0	4.07%
**36**	34.7	33.1	4.61%	34.9	33.4	4.30%
**37**	35.2	33.3	5.40%	35.3	33.6	4.82%
**38**	34.5	32.9	4.64%	34.7	33.2	4.32%
**39**	34.0	32.4	4.71%	33.9	32.5	4.13%
**40**	32.3	31.1	3.72%	32.3	31.3	3.10%

## Conclusion

In this research, aiming at the contradictory problems of insulation, heat dissipation, volume and weight of high voltage power supply under the development trend of high frequency miniaturization and high power, an insulation and thermal conductive composite material with ALSR as the matrix and AlN as the filler is studied to be used for the insulation packaging of output module of high voltage power supply. The main conclusions are drawn as follows:

With increase in the AlN content, the density and thermal conductivity of the composites increase gradually, while the breakdown strength shows gradual weakening. The mutual exclusion relationship between high thermal conductivity and high breakdown strength has been confirmed.

By ANSYS finite element analysis of temperature field, with the increase of the filler fraction, the maximum temperature of the voltage-doubling module drops gradually. When the filler fraction is 40wt%, the maximum temperature decreases to 50°C, which meets the working requirements of the internal electronic components of the voltage-doubling module.

The encapsulation experiment of voltage-doubling module demonstrates that the output voltage keeps unchanged with the increase of the operation time, and the maximum error is 5%. The temperatures measured at 40 points resemble the simulated temperatures in terms of gradient distribution. The maximum experimental error is 6.13%. The practicability of the composite material in the field of insulation and heat conduction packaging has been proved.

## Supporting information

S1 Data (XLSX)
